# Endodontic Reapproach in a Tooth With External Resorption: Case Report

**DOI:** 10.1155/crid/6456051

**Published:** 2025-10-08

**Authors:** Maiara Almeida de Matos, Tais Rocha Donato, Caliandra Pinto Araújo

**Affiliations:** ^1^Endodontics Graduate Program, Professors' Group in Surgery and Implantology, Salvador, Brazil; ^2^Department of General Dentistry, Federal University of Bahia, Salvador, Brazil; ^3^Department of General Dentistry, State University of Feira de Santana, Feira de Santana, Brazil

**Keywords:** cone beam computed tomography, endodontic retreatment, external apical resorption, failure

## Abstract

**Introduction:**

Endodontists face various challenges, and some factors are associated with endodontic treatment failure, such as the presence of resistant microorganisms resulting from inefficient disinfection and nonadhesive coronary sealing. The quality of the execution of the operative protocols, cautious irrigation, and the presence of a hermetic seal from the crown to the apex lead to successful endodontic retreatment.

**Methods:**

This descriptive cross-sectional study approached a female patient, aged 30 years. The data obtained from the literature were collected from PubMed, SciELO, and Google Scholar websites, while the clinical data were obtained from the Teaching Clinic of the Dentistry Postgraduate Department. This study was aimed at reporting a clinical case of conventional endodontic retreatment in Tooth 21 with external apical resorption. Initially, periapical radiographs and cone beam computed tomography were requested from the unit for better assessment and management of the case.

**Results:**

The treatment was carried out with bioceramic cement; in the present case, its use combined with good operative protocols was effective, using simple tools for a successful treatment, ensuring the patient's well-being and quality of life.

**Conclusions:**

The retreatment on Tooth 21, using the existing biological and technological conditions, was effective in generating periodontal health for the associated tissues.

## 1. Introduction

Endodontic treatment is a combination of mechanical and chemical procedures. After removal of pulp tissue and control of microorganisms, the root canal system (RCS) is prepared to receive biocompatible materials that can cover its entire length, guaranteeing an airtight seal. Thus, the root canal can be kept free of microorganisms, restoring the health of periodontal tissues. The keys to successful endodontic treatment are cleaning, shaping, and effective filling [1].

The persistence of microbial infection in the RCS is one of the main causes of endodontic treatment failure. The first resolution option for these cases involves routine endodontic retreatment to overcome the decontamination failure of previous treatment [2].

Root canal disinfection is essential for any endodontic treatment and is a crucial step for effective treatment success [3]. Sodium hypochlorite is still the most used chemical agent in endodontics and is indicated due to its tissue dissolution capacity and antimicrobial activity [4]. As important as the chemical action of the irrigants is the physical action, which is extremely necessary to enhance their cleaning, decontamination, and targeting of all areas of the root canal, including anatomical complexities, flattening, and isthmuses. This physical action is enhanced by different devices, agitating the irrigant via mechanical, sonic, or ultrasonic means or negative pressure. Although highly effective, they are fearfully used in endodontics due to the risk of extravasation of irrigants in teeth with open apexes [5].

Like other dental specialties, endodontics has evolved enormously with the introduction of new technical and scientific resources, and endodontic procedures are becoming more predictable every day. The direct result is that the high success rates of modern endodontic treatments have made it possible to maintain dental health and function far beyond what was possible in the not-too-distant past [6]. Furthermore, with increasing awareness of the importance of preserving teeth, interest in “conventional endodontic retreatment” is also increasing [7].

On the other hand, indications for endodontic retreatment, as a rule, involve complex situations. Obstruction of the canals by dentin shavings, pulp debris resulting from previously performed procedures, ledges, and root deviations, makes access to the foramen difficult. Moreover, the removal of molten metal cores and fiber posts can wear away the tooth structure [8]. According to current literature, root perforations are responsible for approximately 4.2% of cases of extractions of endodontically treated teeth [9]. According to Jurič et al. [10], the clinical success of endodontic therapy is directly related to history of injury, apical periodontitis with large severity, anatomic conditions, use of a bioceramic with poor quality, or an unsatisfactory coronal restoration.

Definitive endodontic therapy in teeth with incomplete root formation or apical root resorption is difficult because the lack of apical constriction makes filling difficult [11]. Also, these teeth may have thin dentin walls and wide canals, making them more susceptible to fractures and complicating the rehabilitation process [12]. Tooth resorption results in the physiological or pathological loss of mineralized tooth tissue, which can be caused by infectious, traumatic, and/or chemical factors and occur locally [13].

MTA-based materials have many qualities that make them advantageous for use in endodontics. These bioceramic materials have antimicrobial properties in their unhardened state. In their fully fixed condition, they present a high degree of biocompatibility and bioactivity. Bioceramics have the ability to stimulate the formation of hard tissue, similar to cementum, when in contact with the periapical tissue [14]. Such desirable properties have expanded the use of these materials to include them in other endodontic treatments, such as perforation repair, pulp therapy, and filling open apical teeth [15].

This study was aimed at presenting a clinical case of endodontic retreatment on Tooth 21 with apical external root resorption.

## 2. Materials and Methods

**Figure 1:**
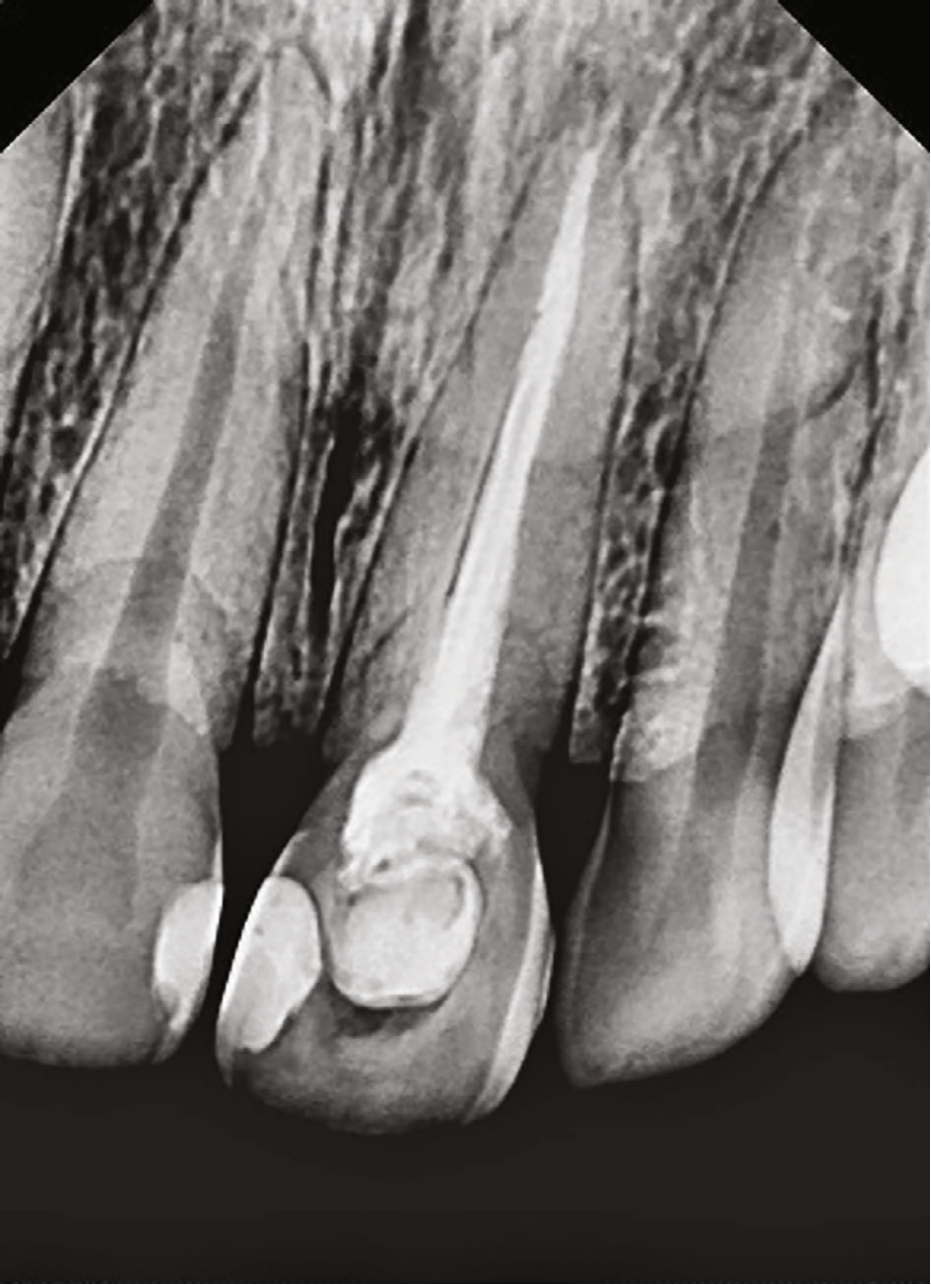
Initial radiograph showing external root resorption on Tooth 21.

**Figure 2:**
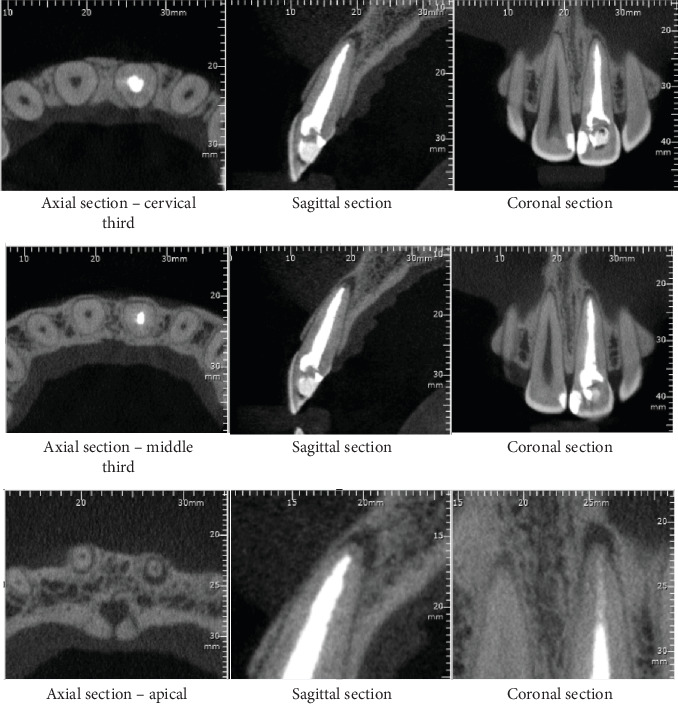
Tomographic sections in the axial, sagittal, and coronal planes of Unit 21 before reintervention.

Patient M.R.S., female, multiracial, 30 years old, visited the teaching clinic of Dentistry Postgraduate Department in Salvador, Bahia, Brazil, for dental care, with a history of pain in Tooth 21 and aesthetic complaints due to its discoloration. The patient's medical history did not reveal any relevant pathology. The patient reported having undergone previous endodontic treatment in a single session 2 years before. Over time, this same tooth became sensitive and symptomatic upon palpation. The radiographic examination showed that Tooth 21 had apical external root resorption (Figure 1). For better management and confirmation of the diagnosis, a cone beam computed tomography (CBCT) was also performed, which confirmed the apical reabsorption, according to the axial, coronal, and sagittal planes (Figure 2). After the examinations and medical history survey, a clinical diagnosis of acute apical periodontitis was reached, with indication of endodontic retreatment.

The clinical examination revealed healthy gingiva, with no cavities, considering good oral health conditions. Moreover, the pulp chamber was filled with definitive sealing material. The cold pulp sensitivity test had a negative response. On the other hand, vertical and horizontal percussion tests were present, whereas the assessment of tooth mobility was absent. In addition, quantitative evaluation of preoperative and postoperative periodontal probing depths demonstrated uniform measurements of 3 mm at the mesial, middle third, and distal aspects (Table 1).

In the first consultation, infiltrative anesthesia was performed in the region of the anterior alveolar nerve with 4% articaine with 1:100,000 epinephrine (DFL, Rio de Janeiro) on Tooth 21. Soon after, absolute isolation was performed using a rubber sheet (Mandeitex, São José dos Campos). Coronary access was performed with the 1014 spherical diamond tip (KG, Sorensen, São Paulo) and Endo Z (AllPrime, Brazil). In addition, 1/3 of the cervical gutta-percha (Dentsply Sirona, Maillefer, Switzerland) was removed with a Gates #2 drill (MK Life, Brazil). Furthermore, the cervical, middle, and apical thirds were cleared with a 25-mm Reciproc #25 reciprocating file (VDW, Brazil) and 25-mm Hedstroem files up to #40 (Dentsply Sirona, Maillefer, Switzerland) to remove canal side wall materials. Then, the root canal was reprepared with a #40 X1 25-mm reciprocating file (MK Life, Brazil). The irrigation protocol was 2.5% sodium hypochlorite (3 cycles of 20 s), followed by 17% EDTA (3 cycles of 20 s), ending with 2.5% sodium hypochlorite (3 cycles of 20 s). During the entire process, the substances were stirred for 3 min.

**Figure 3:**
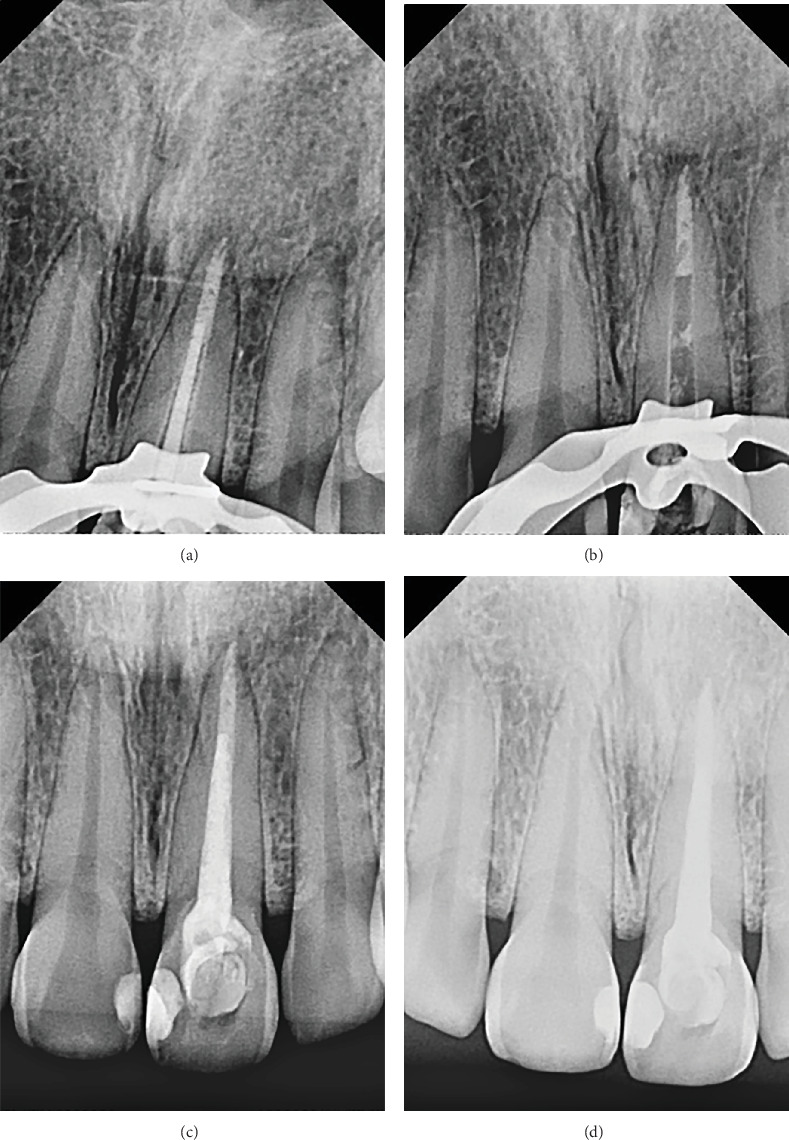
(a) Cone test. (b) MTA buffer. (c) Final radiograph. (d) One-month maintenance. Source: the author.

Lastly, the apical plug was made with MTA, which was manipulated on a polished glass plate (Golgran, São Caetano do Sul, São Paulo), with a No. 24 manipulation spatula (Golgran, São Caetano do Sul, São Paulo). Dispensing the first drop of liquid (distilled water) and a measure of the dosing paddle, the powder was added little by little to the liquid, which was manipulated for 30 s, with the help of a 1.2-mm MTA applicator (Angelus, Londrina, Brazil). Using a No. 2 Paiva presser, it reached the apical middle third (Golgran, Brazil) (Figure 3b). Soon after, the cone test was carried out, which consisted of calibrating the gutta-percha 60 (Dentsply Sirona, Maillefer, Switzerland), using the main M cone, two FM accessories, and a calibrating ruler (Angelus, Londrina, Paraná) (Figure 3a).

Thus, the filling was performed with nonresin bioceramic cement BIO-C SEALER (Angelus, Londrina, Brazil). Then, the gutta-percha was cut 2 mm below the root canal entrance, cleaning the pulp chamber with cotton balls and alcohol. In addition, the definitive restoration was performed with 37% phosphoric acid etchant (Allprime, Brazil) for 15 s, and the cavity was irrigated and dried. After this, the adhesive system (Single Bond 2, Germany) was applied in a single step with photoactivation of the cavity for 20 s and, finally, the form resin (Ultradent, São Paulo, Brazil) was infused into the cavity.

An x-ray was performed to completely verify the obturation of the root canal and subsequent clinical and radiographic monitoring of the endodontic therapy (Figure 3c). Finally, improvement was observed after just over a month (Figure 3d).

**Figure 4:**
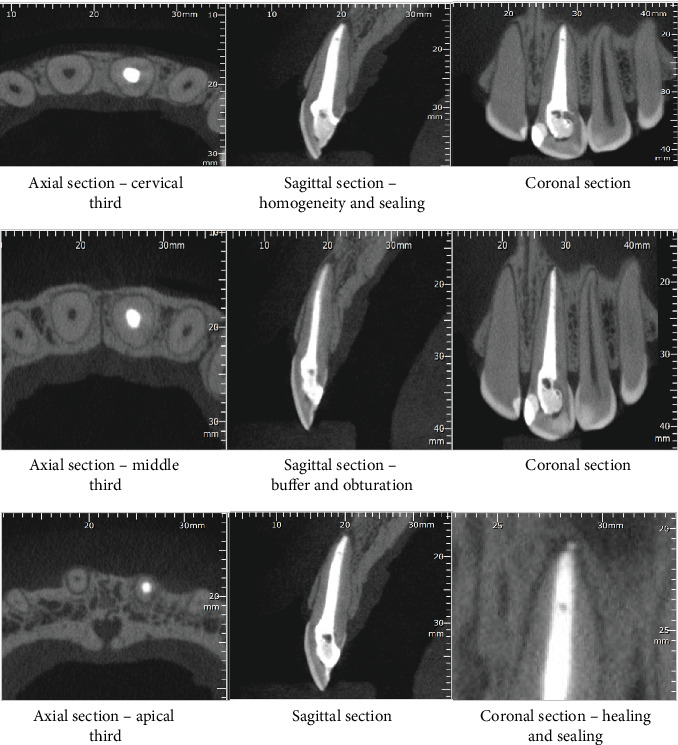
Tomographic sections in the axial, sagittal, and coronal planes of Unit 21 after reintervention.

The patient was advised to maintain oral health and follow up on the case. After 4 months, she performed a CBCT of the treated unit, revealing tissue recovery and absence of inflammation (Figure 4). Further clinical details are available in Appendices S1, S1.1, S2, and S3.

## 3. Discussion

Failure in endodontics is often detected by routine radiographs, which indicate the maintenance or progression of periapical pathological processes. The European Society of Endodontology suggests that follow-up x-rays be taken at least 1 year after completion of endodontic therapy to confirm the health of the periodontal space around the tooth [16]. This case report found that the previous treatment failure may have been caused by several factors, such as heterogeneous filling in the root canal and remaining infection after closing the canal.

According to the literature, the success rate of endodontic retreatment ranged from 40% to 85%, although most of this work was carried out more than 20 years ago. Current endodontic techniques are quite different from those used in the past [17]. Recent scientific studies demonstrate that the success rate of dental retreatment has increased significantly, reaching a level of 95% [6].

The anatomical complexity of the RCS makes it impossible to remove with mechanical instruments all organic and inorganic content, whether contaminated or not. In this line of thought, irrigation plays an essential role in reaching confined areas that are difficult to access by manual or mechanized preparation [18]. Hence, the quality of cleaning and disinfection achieved with chemical agents during endodontic therapy is directly related to the success of the treatment, as it significantly reduces the number of microorganisms [19].

The 2.5% NaOCl solution offers advantages in the chemical–mechanical preparation of RCSs and is therefore recommended for routine use. However, it is important to highlight that in case of persistent lesions or prolonged tooth exposure to the pulp chamber of the oral cavity, usually with a high level of contamination, the effect of the irrigant must be enhanced through agitation, association with other chemical substances, or intracanal medications [20]. In the present case, the irrigation protocol began with 2.5% sodium hypochlorite (3 cycles of 20 s), followed by 17% EDTA (3 cycles of 20 s), ending with 2.5% sodium hypochlorite (3 cycles of 20 s). Kashikar et al. reported that this protocol is sufficient to combat infections and mechanically and chemically remove microorganisms, dentin scrapings, pulp tissue, and other complications [21].

The effectiveness of the protocol is associated with the enhancement of substances through their agitation, which heats, moves, and suspends debris from the RCS, increasing its cleaning and consequently ensuring a successful treatment. Agitation options include conventional, mechanical, sonic, ultrasonic, and EndoVac systems (Sybron, Orange, United States). The present study used mechanical agitation with sodium hypochlorite and ethylenediaminetetraacetic acid.

One of the reasons that supports the great need for retreatment monitoring is the need for a good coronary seal after therapy, a key factor that is often absent [22]. Filling canals directly exposed to saliva (presence of caries, infiltration of the apical barrier, and damage to the tooth material or structure) poses a significant risk of recontamination [23]. Failure of appropriate endodontic therapy may be due to microbial factors (intra and extraradicular infections) or endogenous (presence of cysts, tissue healing, etc.) and exogenous (foreign body resulting from endodontic materials) nonmicrobial factors [24]. In this case report, an incomplete apical sealing was identified before retreatment, being the possible cause of periodontitis, due to the lack of sealing in the resorbed region. MTA (Angelus, Londrina, Brazil) was used as a buffer to remedy this flaw and hermetically seal the apical region, as this material has excellent biological properties, promoting good adaptation, antimicrobial activity, biocompatibility, and bioactivity. Furthermore, the bioceramic cement Bio-C Sealer (Angelus, Londrina, Brazil) was applied to the filling of the middle and cervical thirds in association with the calibrated main gutta-percha, as this cement also has good sealing capacity and a favorable setting time, biocompatibility, and ease of manipulation and insertion.

The patient's medical history survey and radiological studies were aimed at providing the best appropriate treatment for the patient, such as requesting high-resolution computed tomography [25]. The CBCT confirmed that Element 21 in the case in question had apical external reabsorption, thus demonstrating that the technological resources used were crucial to the success of retreatment, adding essential information.

It is worth noting that external root resorption may originate from different causes, such as trauma or infection, and that these distinct etiologies influence not only the speed of progression but also the choice of treatment. Resorptions of traumatic origin, which often result from damage to the periodontal ligament and cementum, tend to evolve rapidly and demand immediate and specific management. On the other hand, inflammatory resorptions—usually linked to microbial contamination—are more responsive to protocols based on disinfection and endodontic retreatment [26]. This differentiation is clinically relevant because it directly impacts the expected prognosis. However, when such observations are based solely on isolated clinical cases, caution is warranted. The limitations inherent in case reports—such as the absence of control groups and restricted external validity—must be clearly acknowledged [27]. Therefore, while they can guide individual decisions, more robust conclusions require support from studies with stronger methodological designs.

Factors that lead to external root resorption include persistent stimuli that cause inflammation and subsequent resorption, such as trauma, excessive orthodontic movement, chronic periapical lesions, and/or multifactorial diseases [28]. The study by Silva et al. recommends endodontic treatment/retreatment to remove the causative agent and, if necessary, multidisciplinary treatment involving other specialties [29].

## 4. Conclusions

In this study, the retreatment on Tooth 21, using the existing biological and technological conditions, was effective in generating periodontal health for the associated tissues. Therefore, careful protocols and biocompatible materials appear to be powerful and simple tools for successful endodontic treatment.

## Figures and Tables

**Table 1:** Periodontal examination.

**Probing depths (mm)**	**Mesial**	**Middle third**	**Distal**
Preoperative	3 mm	3 mm	3 mm
Postoperative	3 mm	3 mm	3 mm

## Data Availability

The data used to support the findings of this study are available from the corresponding author upon request.

## Supporting Information

Supporting information Additional supporting information can be found online in the Supporting Information section. The supporting information is provided in Appendices S1, S1.1, S2, and S3, which contain additional details about the clinical and radiography procedures. This information supports the case description presented in the manuscript.
