# Supplementary material for: Endodontic Reapproach in a Tooth With External Resorption: Case Report
Source: Case Rep Dent. 2025 Oct 8;2025:6456051. doi: 10.1155/crid/6456051 (PMC12527599; doi:10.1155/crid/6456051)
Supplement: Supporting information — Additional supporting information can be found online in the Supporting Information section. The supporting information is provided in Appendices S1, S1.1, S2, and S3, which contain additional details about the clinical and radiography procedures. This information supports the case description presented in the manuscript. [file 6456051.f1.zip › Appendix B - IRB CBCT MirianRochadeSouzaCunha-374927.pdf]

IMAGEM PIERRE FAUCHARD
Radiologia buco-maxilo-facial

Central de Atendimento: (71) 3358-06

Dr. Sérgio Freitas Ribeiro
CRO-BA 5104

WhatsApp (71) 99951-4466

www.IMAGEMPIERRE.com.br

PACIENTE: Mirian Rocha de Souz Cunha

SOLICITANTE: Hellen Mayara

E-MAIL: examepaciente@seianucleo.com.br

DATA: 16/10/2023

ENVIAR POR: (MARCAR OPÇÃO)

E-MAIL | CD | PASTA PLÁSTICA | PAPEL FOTOGRÁFICO | ACETATO | PRECISO DE BLOCOS DE REQUISIÇÃO

TOMOGRAFIA CONE BEAM

REGIÃO DENTOALVEOLAR

- [x] MAXILA
- [ ] MANDÍBULA
- [ ] TÉCNICAS DE LOCALIZAÇÃO:
  - [ ] Retidos
  - [ ] Supranumerários
  - [ ] Corpo Estranho
- [ ] PESQUISA DE:
  - [ ] Fraturas
  - [ ] Condutos
  - [ ] Corticais
- [ ] CIRURGIA GUIADA (IMAGEM PIERRE)
  - [ ] PROTOCOLO
  - [ ] REGIÃO INDIVIDUAL
  - DENTES
- [ ] SELEÇÃO INDIVIDUAL

18	17	16	15	14	13	12	11	21	22	23	24	25	26	27	28
48	47	46	45	44	43	42	41	31	32	33	34	35	36	37	38

FACE COMPLETA

- [ ] ATM
  - [ ] DIREITA
  - [ ] ESQUERDA
- [ ] SEIOS MAXILARES
- [ ] VIAS AÉREAS
- [ ] ORTOGNÁTICA
- [ ] ORTODONTIA

COMO DESEJO RECEBER A TOMOGRAFIA:

DICOM POR EMAIL | CD CS IMAGING | Película Radiográfica | Papel Fotográfico

MODELOS

- [ ] ESCANEAMENTO INTRAORAL (HD)
  - [ ] CONVENCIONAL
    - [ ] STL
    - [ ] MODELO DIGITAL
  - [ ] INVISALIGN
  - [ ] OUTROS ALINHADORES

MODELOS DIGITAIS SÃO MAIS SEGUROS E NÃO OCUPAM ESPAÇO FÍSICO.

- [ ] Ortodôntico
  - [ ] IMPRESSO 3D
  - [ ] IMPRESSO 3D REFORÇADO

- [ ] Análise Calcográfica

>>>> FINALIDADE DOS EXAMES E OBSERVAÇÕES <<<<

Solicito Tomografia cone Beam em alta Resolução e FOV Pequeno do un 21 para diagnostico de endodontia avaliando se pode haver trinca ou fratura
Por favor, enviar o Dicom

Escrever aqui as informações que considere relevantes para realização dos procedimentos.

NÚCLEO PÓS-GRADUAÇÃO EM ODONTOLOGIA

NÚCLEO PÓS-GRADUAÇÃO EM ODONTOLOGIA

EXAMES RADIOGRÁFICOS EXTRA BUCAIS

PANORÂMICA DIGITAL

- ☐ Topo
- ☐ Oclusão
- ☐ Implantes — REGIÕES

- ☐ P.A. DE MANDÍBULA
- ☐ P.A. DE SEIO MAXILA
- ☐ MÃO E PUNHO (Idade Óssea)
- ☐ ATM NORMA LATERAL (Boca aberta e máxima intercuspidação)

☐ TELERADIOGRAFIA SEM TRAÇADO

☐ FRONTAL ☐ LATERAL ☐ 45º

DOCUMENTAÇÃO ORTODÔNTICA

FOTOS EXTRA BUCAIS	FOTOS INTRA BUCAIS
☐ P. Direito ☐ Frontal	☐ Frontal ☐ Perfil Direito
☐ P. Esquerdo ☐ Sorriso	☐ Perfil Esquerdo ☐ Arcos
OUTRAS:	OUTRAS:

☐ TELERADIOGRAFIA COM ANÁLISE COMPUTADORIZADA:

☐ ADENÓIDE (Via Nasofaríngea)	☐ PROFIS	☐ JARABAK
☐ USP/UNICAMP	☐ RICKETTS 32 FATORES	☐ UNICAMP
☐ DOWNS	☐ STEINER	☐ USP
☐ IBEO	☐ TWEED	☐ VIAS AÉREAS
☐ LAVERGNE/PETROVIC	☐ MCNAMARA	☐ ORTOGNÁTICA

☐ OUTRA: ______________________________

EXAMES RADIOGRÁFICOS INTRA BUCAIS

PERIAPICAIS DIGITAIS (PARALELISMO)

- ☐ Arcos Dentários (Boca Completa)
- ☐ Dentes Assinalados

			55	54	53	52	51	61	62	63	64	65			
18	17	16	15	14	13	12	11	21	22	23	24	25	26	27	28
48	47	46	45	44	43	42	41	31	32	33	34	35	36	37	38
			85	84	83	82	81	71	72	73	74	75			

INTERPROXIMAIS DIGITAIS (BITE WING)

- ☐ Direito
- ☐ Esquerdo
- ☐ Região de: ______________________

OCLUSAIS

☐ Superior ☐ Inferior

ENDEREÇOS E CONVÊNIOS

ESCANEIE O QR CODE ABAIXO PARA VISUALIZAR OS ENDEREÇOS DAS NOSSAS UNIDADES:

ESCANEIE O QR CODE ABAIXO PARA ACESSAR O NOSSO SITE:

PRINCIPAIS CONVÊNIOS

PETROBRÁS
BRADESCO
REDE UNNA
PORTO SEGURO
SESI
CAMED
PLAS / JMU
UNAFISCO
SULAMÉRICA
POSTAL SAÚDE
BANCO CENTRAL
SAÚDE CAIXA
AMIL
ODONTOSERV
BB DENTAL

PARA OUTROS CONVÊNIOS, ENTRAR EM CONTATO PARA VERIFICAR ATENDIMENTO.

Studio 3 - Gestão Empresarial
Clinica Imagem Pierre Fauchard

Ordem de Serviço: ITA. 374927 de 09/11/2023 10:47:10

Clinica Imagem Pierre Fauchard
Cliente: 116123. Mirian Rocha de Souza Cunha
Idade: 30a 8m **Género:** Feminino
O.S. Nº: ITA 374927 **Data:** 09/11/2023
Dentista Dr(a): Núcleo pós graduação em odontologia

Cliente

Mirian Rocha de Souza Cunha (Cod.: 116123)
CPF: 056.865.545-10
Nascimento: 25 mar 1993 - Idade: 30 anos e 8 meses

Endereço (Residencial)
.
-
Tel: (Celular) (71) 99698-4421
e-mail: mirianrochafisio@gmail.com

Cliente RETIRA

Dentista

Núcleo pós graduação em odontologia
CRO:

Endereço ()

Tel:
e-mail: examepaciente@sejanucleo.com.br

PACIENTE EXTRA

Comentários
JOICE KELLY

Convênio: 2. Particular

EX	QTD	Serviço/Grupo ou Item	Detalhe	Rep.	Valor Convênio	Valor Cliente
☐	1	37. Tomografia 01 Sextante			R$ 0,00	R$ 184,00
		**Totais a receber:**			**R$ 0,00**	**R$ 184,00**

Forma(s) de Recebimento(s)

Nome	Valor
Cartão de Crédito REDE	R$ 184,00

Convênio: R$ 0,00
Receb.: **Previsto** **Particular:** R$ 184,00

Abertura: 09/11/2023 10:47 **Entrega prevista:** 16/11/2023 15:00
Entregue:
