# Supplementary material for: Endodontic Reapproach in a Tooth With External Resorption: Case Report
Source: Case Rep Dent. 2025 Oct 8;2025:6456051. doi: 10.1155/crid/6456051 (PMC12527599; doi:10.1155/crid/6456051)
Supplement: Supporting information — Additional supporting information can be found online in the Supporting Information section. The supporting information is provided in Appendices S1, S1.1, S2, and S3, which contain additional details about the clinical and radiography procedures. This information supports the case description presented in the manuscript. [file 6456051.f1.zip › Appendix A -PatientConsent_signed.pdf.pdf]

WILEY

Patient Consent Form

To record a patient's consent to publication of information relating to them or a relative, in a Wiley publication.

Name of patient: MIRIAN ROCHA DE SOUZA CUNHA

Title of publication/product: ENDODONTIC REAPPROACH IN A TOOTH WITH E

Principal author/editor: MAIARA ALMEIDA DE MATOS

Principal author/editor's address: SALVADOR, BA - BRAZIL

I, MIRIAN ROCHA DE SOUZA CUNHA (the "Licensor"), give my permission to use clinical information/video/photographic material relating to MIRIAN ROCHA in the publication identified above to be published by John Wiley & Sons, Inc. or one of its affiliated companies ("Wiley"), such permission to extend to publication of the information by Wiley and its licensees in the publication and all related derivative and ancillary works, in all media and languages now or hereafter known, throughout the world.

In cases where the patient has died or is incapable of giving consent, consent may be given by the next of kin. If the patient is under the age of 16, consent should be given by a parent or guardian.

I understand that:

(1) The patient's name will not be published. However I understand that there is a possibility that I may be identified from the clinical information and identifiable material (such as videos/photographic material) for which permission is granted hereunder.

(2) I understand that my image may be used on the cover of the publication. Yes ☑ No ☐

(3) If the publication or any related derivative and ancillary work is published on an open access basis, I understand that it may be accessed freely throughout the world.

The laws of the country set forth below shall apply to this agreement according to your country of residence at the time of signing, without regard to conflicts of law rules. The corresponding jurisdiction shall be the forum for adjudication of all disputes arising in connection with this agreement:

Country	Applicable Law	Agreed Jurisdiction
United States and all other countries not expressly stated herein	State of New York	New York, NY
United Kingdom and EMEA (excluding Germany)	England and Wales	England and Wales
Germany	Federal Republic of Germany	Weinheim
Australia	State of Victoria	Melbourne
Singapore	Singapore	Singapore
India	Where contracted by Wiley India Pvt Ltd: State of Delhi, India<br>Where contracted by other Wiley entities: Singapore	New Delhi, India.<br>Arbitration in Singapore administered by the Singapore International Arbitration Centre (SIAC) in accordance with the Arbitration Rules of SIAC for the time being in force. The language of the arbitration shall be English. The decision of the arbitrator shall be final and may be used as a basis for judgement in any country.
Japan	Where contracted by Wiley Publishing Japan KK: Japan<br>Where contracted by other Wiley entities: Singapore	Arbitration in Tokyo under the Rules of Arbitration of the International Chamber of Commerce by one or more arbitrators appointed in accordance with the said Rules. The language of the arbitration shall be English. The decision of the arbitrator shall be final and may be used as a basis for judgement in any country.
All Asia Pacific (excluding Australia, India and Singapore)	Singapore	Arbitration in Singapore administered by the Singapore International Arbitration Centre (SIAC) in accordance with the Arbitration Rules of SIAC for the time being in force. The language of the arbitration shall be English. The decision of the arbitrator shall be final and may be used as a basis for judgement in any country.

SIGNATURE OF PATIENT/PARENT/GUARDIAN/NEXT OF KIN

IF PARENT/GUARDIAN/NEXT OF KIN, STATE RELATIONSHIP TO PATIENT

SALVADOR, BA - BRAZIL

2023/11/21

SIGNATURE OF HEALTH PROFESSIONAL OBTAINING PERMISSION (IF APPROPRIATE)

SALVADOR, BA - BRAZIL

2023/11/21

Note to principal author: The original signed consent form should be retained by the principal author.

Note to health professional: In addition to the consent form, please ensure that any other necessary permissions are cleared for use of the information, including any permissions required for use of information contained in medical records.
