# Supplementary figures and images for: Endodontic Reapproach in a Tooth With External Resorption: Case Report
Source: Case Rep Dent. 2025 Oct 8;2025:6456051. doi: 10.1155/crid/6456051 (PMC12527599; doi:10.1155/crid/6456051)

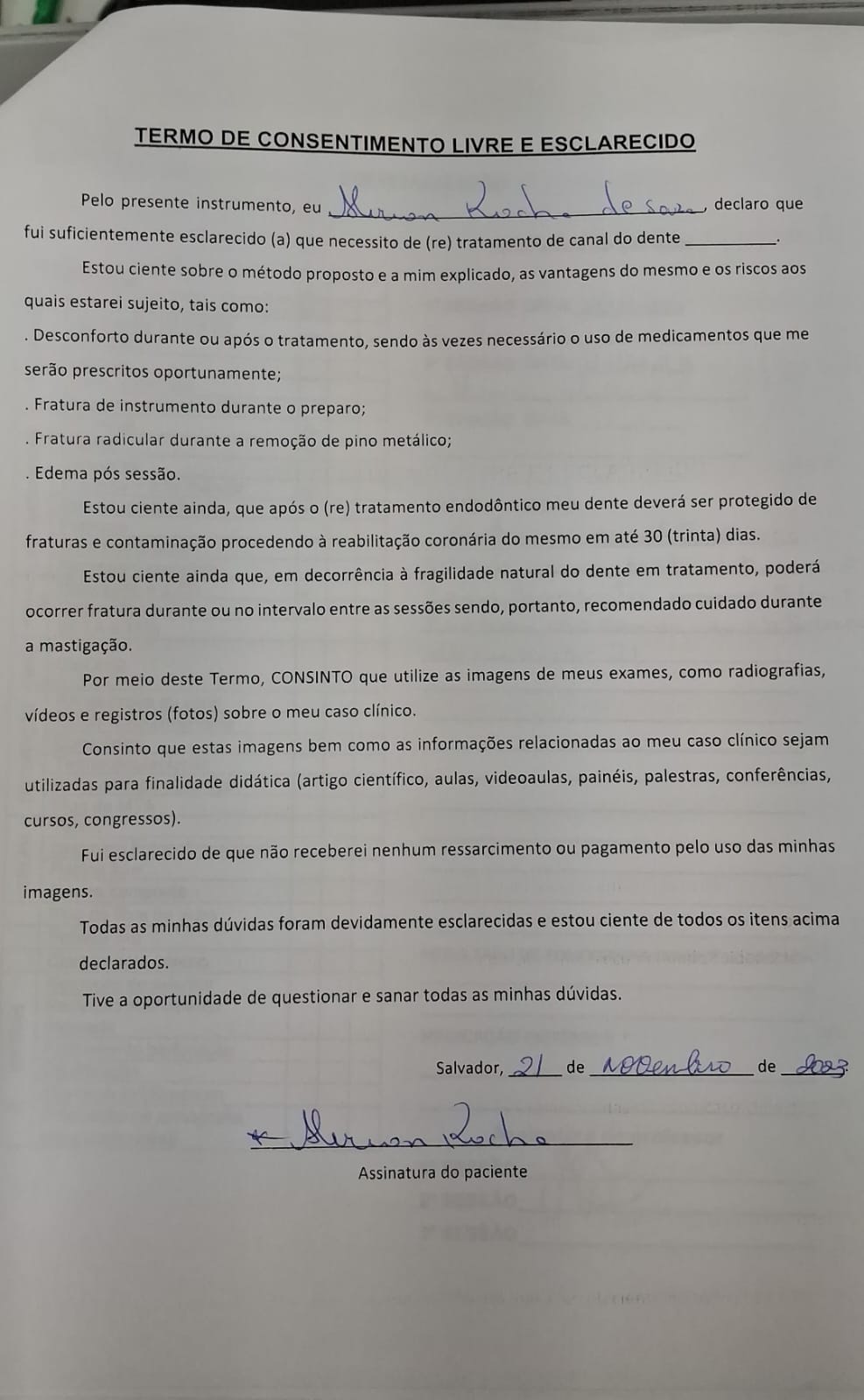

Supplement: Supporting information — Additional supporting information can be found online in the Supporting Information section. The supporting information is provided in Appendices S1, S1.1, S2, and S3, which contain additional details about the clinical and radiography procedures. This information supports the case description presented in the manuscript. [file 6456051.f1.zip › Appendix A.1 - Patient Consent Form_Portuguese.jpg]
